# Impact of COVID-19 outbreak on regional STEMI care in Germany

**DOI:** 10.1007/s00392-020-01703-z

**Published:** 2020-07-16

**Authors:** Karl Heinrich Scholz, Björn Lengenfelder, Christian Thilo, Andreas Jeron, Stefan Stefanow, Uwe Janssens, Johann Bauersachs, P. Christian Schulze, Klaus Dieter Winter, Jörg Schröder, Jürgen vom Dahl, Nicolas von Beckerath, Karlheinz Seidl, Tim Friede, Thomas Meyer

**Affiliations:** 1 Department of Cardiology, Medizinische Klinik I, St. Bernward Hospital, Treibestraße 9, 31134 Hildesheim, Germany; 2 Department of Cardiology, University of Würzburg and Comprehensive Heart Failure Center Würzburg, Medizinische Klinik und Poliklinik I, Oberdürrbacher Straße 6, 97080 Würzburg, Germany; 3 Department of Internal Medicine I, Division of Cardiology, University Hospital of Augsburg, Stenglinstr.2, 86156 Augsburg, Germany; 4 Department of Cardiology, Rems-Murr-Kliniken GmbH, Am Jakobsweg 1, 71364 Winnenden, Germany; 5 Department of Cardiology, Klinikum Ludwigsburg-Bietigheim gGmbH, Posilipostraße 4, 71640 Ludwigsburg, Germany; 6 Department of Cardiology, St. Antonius Hospital Eschweiler, Dechant-Deckers-Str. 8, 52249 Eschweiler, Germany; 7 Department of Cardiology, Medizinische Hochschule Hannover, Carl-Neuberg-Str.1, 30625 Hannover, Germany; 8 Department of Internal Medicine I, Division of Cardiology, University Hospital Jena, Am Klinikum 1, 07740 Jena, Germany; 9 Department of Cardiology, Hermann-Josef-Hospital Erkelenz, Tenholter Str. 43, 41812 Erkelenz, Germany; 10 Department of Cardiology, University of Aachen, Medizinische Klinik I, Pauwelsstraße 30, 52074 Aachen, Germany; 11 Department of Cardiology, Kliniken Maria Hilf GmbH Mönchengladbach, Viersener Strasse 450, 41063 Mönchengladbach, Germany; 12 Department of Cardiology, Allgemeines Krankenhaus Viersen GmbH, Hoserkirchweg 63, 41747 Viersen, Germany; 13 Department of Cardiology, Medizinische Klinik I, Klinikum Ingolstadt, Krumenauerstraße 25, 85049 Ingolstadt, Germany; 14 Department of Medical Statistics, University Medical Center Göttingen, University of Göttingen, and German Center for Cardiovascular Research (DZHK), Partner Site Göttingen, Humboldtallee 32, 37073 Göttingen, Germany; 15 Department of Psychosomatic Medicine and Psychotherapy, University Medical Center Göttingen, University of Göttingen, and DZHK, Partner Site Göttingen, Waldweg 33, 37073 Göttingen, Germany

**Keywords:** ST-segment elevation myocardial infarction (STEMI), Percutaneous coronary intervention (PCI), Key quality indicators, Contact-to-balloon time, Mortality, COVID-19

## Abstract

**Aims:**

To assess the impact of the lockdown due to coronavirus disease 2019 (COVID-19) on key quality indicators for the treatment of ST-segment elevation myocardial infarction (STEMI) patients.

**Methods:**

Data were obtained from 41 hospitals participating in the prospective Feedback Intervention and Treatment Times in ST-Elevation Myocardial Infarction (FITT-STEMI) study, including 15,800 patients treated for acute STEMI from January 2017 to the end of March 2020.

**Results:**

There was a 12.6% decrease in the total number of STEMI patients treated at the peak of the pandemic in March 2020 as compared to the mean number treated in the March months of the preceding years. This was accompanied by a significant difference among the modes of admission to hospitals (*p* = 0.017) with a particular decline in intra-hospital infarctions and transfer patients from other hospitals, while the proportion of patients transported by emergency medical service (EMS) remained stable. In EMS-transported patients, predefined quality indicators, such as percentages of pre-hospital ECGs (both 97%, 95% CI = − 2.2–2.7, *p* = 0.846), direct transports from the scene to the catheterization laboratory bypassing the emergency department (68% vs. 66%, 95% CI = − 4.9–7.9, *p* = 0.641), and contact-to-balloon-times of less than or equal to 90 min (58.3% vs. 57.8%, 95%CI = − 6.2–7.2, *p* = 0.879) were not significantly altered during the COVID-19 crisis, as was in-hospital mortality (9.2% vs. 8.5%, 95% CI = − 3.2–4.5, *p* = 0.739).

**Conclusions:**

Clinically important indicators for STEMI management were unaffected at the peak of COVID-19, suggesting that the pre-existing logistic structure in the regional STEMI networks preserved high-quality standards even when challenged by a threatening pandemic.

**Clinical trial registration:**

NCT00794001

**Electronic supplementary material:**

The online version of this article (10.1007/s00392-020-01703-z) contains supplementary material, which is available to authorized users.

## Introduction

According to a well-established recommendation in the current guidelines, patients presenting with ST-segment elevation myocardial infarction (STEMI) require instant revascularization by percutaneous coronary intervention (PCI), since any time delay in treatment is associated with increased cardiac damage and elevated mortality [1]. Given the overall high morbidity and mortality in STEMI patients, systems of STEMI care have been established to expedite PCI workflows to minimize the ischaemic time from symptom onset to balloon inflation during PCI. As shown recently, predefined key quality indicators for STEMI care have remained stable over the last decade having been successfully implemented in local STEMI care networks before being challenged by a globally occurring crisis [2]. However, the extent to which a pandemic like the currently ongoing corona virus disease 19 (COVID-19) affects the health care system for STEMI treatment is largely unknown [3, 4]. Due to the rapid spread of the corona virus across the globe since its initial outbreak in Wuhan, China, in December 2019, the pandemic has placed enormous strain on nearly all healthcare systems worldwide [5–7]. Since most countries confronted with the novel betacoronavirus SARS-CoV-2 implemented stringent infection control measures, these restrictions may have a profound impact on routine medical care and, in particular, STEMI management [3, 4, 7–15]. In this paper, we assess the impact of the global COVID-19-induced lockdown on STEMI care with a particular focus on key quality indicators using data from the ongoing, multicentre, prospective Feedback Intervention and Treatment Times in ST-Elevation Myocardial Infarction (FITT-STEMI) study.

## Methods

### Design of the FITT-STEMI study

The FITT-STEMI trial, which currently recruits more than 10% of all STEMI cases in Germany [16], was initially designed to prospectively assess the prognostic benefit of systematic and formalized data assessment and interactive feedback on time to interventional treatment for patients presenting with STEMI. The primary aim of the FITT-STEMI study, registered at clinicaltrials.gov under the number NCT00794001, was to implement standardized feedback-driven quality management for timely reperfusion therapy in existing regional STEMI care networks. The study protocol was approved by the ethics committee of the Medical Faculty at the University of Göttingen (1/10/07) and by the local ethics committee of each participating PCI centre. Details on the study design, data collection, and outcome measures including first clinical results have been described [17, 18].

### Data assessment and outcome measures

The participating 41 PCI hospitals are all tertiary care centres included seven university clinics. All centres collected detailed clinical information for each consecutive STEMI patient on treatment times, including time intervals from symptom onset to arrival of the emergency medical service (EMS) on the scene, the duration of out-of-hospital treatment and the transport time to the nearest PCI centre as well as intra-hospital times for the transfer to the catheterization laboratory and the time to balloon inflation. In addition, comorbid diagnoses, medical history, Thrombolysis in Myocardial Infarction (TIMI) risk score for STEMI [19], medical history, cardiac risk factors, and results from coronary angiography as well as the PCI procedure were documented on a case-report form. The following predefined key quality data for STEMI management were obtained: percentages of pre-hospital ECG recordings, pre-announcement of STEMI diagnosis by telephone before arrival at the hospital, direct transfer to the catheterization laboratory bypassing the emergency department, and contact-to-balloon time equal or less than 90 min.

### Statistical analysis

**Fig. 1:**
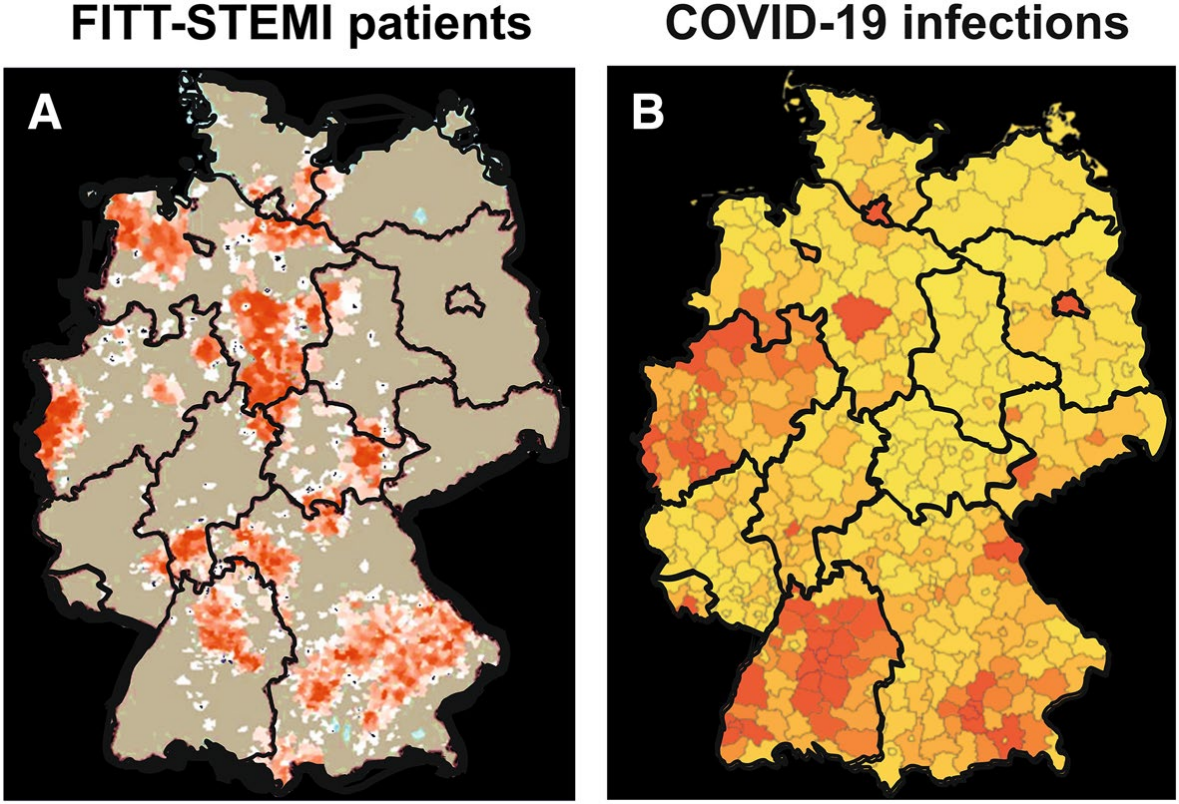
Catchment area maps showing home postcodes for all STEMI patients included in the FITT-STEMI study (**a**) and regional hot spots of the COVID-19 outbreak during the initial phase of the epidemic in Germany (**b**). The maps depict the overlap of the two distributions particularly in the Lower Rhine area in North-West Germany

For this post hoc study of the influence of the COVID-19-induced lockdown on German regional STEMI care networks, raw data from January 2017 to inclusively March 2020 were analyzed. Descriptive statistics were calculated as means with standard errors of the mean for continuous variables and frequencies with percentages for categorical variables. Continuous data were compared using Student´s *t* test between the two groups of patients treated in March 2020 versus the three March months in the preceding years 2017–2019. Categorical variables from these two groups were analyzed using chi-square tests. For some of the analyses, the study cohort was classified along the guideline-recommended threshold for a contact-to-balloon time equal or less than 90 min. A series of linear regression models were computed with intervals of treatment times as dependent variable and study recruitment in March 2020 during the corona virus outbreak versus the March months in the three preceding years as independent variable. These models were adjusted for gender, age, TIMI risk score, infarct localization, and thrombolytic therapy. Logistic regression models for key quality markers in STEMI care as independent, dichotomous variables were created using the same set of covariables. In addition, a subgroup analysis was performed for all FITT-STEMI clinics in the Lower Rhine area around Heinsberg located close to Aachen, because this region in North Rhine-Westphalia was most heavily affected by the SARS-CoV-2 outbreak. As shown in the catchment area maps in Fig. 1, the recruitment of patients to the nationwide FITT-STEMI study centres partially overlapped with areas of high prevalence of confirmed SARS-CoV-2-positive cases, specifically at the Lower Rhine area. Statistical analyses were performed using the SAS system (version 9.4). All reported *p* values are two-sided, and *p* values < 0.05 were considered statistically significant. No formal adjustment for multiple testing was carried out.

## Results

### Number of STEMI patients during the peak COVID-19 lockdown

In total, 15,800 consecutive STEMI patients presenting within 24 h of symptom onset were treated from January 1, 2017, to March 31, 2020 in all participating centres of the FITT-STEMI study group. Of those, the majority of patients (*n* = 12,070, 76.4%) were transported directly from the field to the PCI hospital by EMS (Table 1). Transfers from other, non-PCI-capable hospitals to FITT-STEMI clinics constituted the second largest group with 1733 patients (11.0%), which was followed by the group of STEMI patients arriving at hospitals independently as self-referrals (*n* = 1598; 10.1%). During the total study period starting from January 2017, the average number of patients treated monthly who had a documented myocardial infarction experienced during hospital stay was ten patients (2.5%). Among all STEMI patients treated, 122 patients had pre-PCI fibrinolysis (0.8%) and 14,186 patients received primary PCI (89.8%). A detailed description of the total study population, including the comparison between the two groups of STEMI patients treated in March 2020 and the preceding three March months in the years 2017–2019, is given in Table 1.

**Table 1:** Baseline characteristics of the total study cohort of STEMI patients treated from January 2017 to March 2020 in 41 German hospitals participating in the FITT-STEMI trial

	Total study cohort (*n* = 15,800)	Patients included in the March months 2017/2018/2019 (*n* = 1329)	Patients included in March 2020 (*n* = 387)	*P* value
Demographic data				
Male gender (*n* = 15,800)	11,558 (73%)	953 (72%)	277 (72%)	0.960
Age ± SD (years) (mean, SEM) (*n* = 15,415)	63.9 ± 0.1	63.6 ± 0.4	64.5 ± 0.7	0.226
Transport routes				
EMS	12,070 (76.4%)	1008 (75.8%)	320 (82.7%)	0.017
Inter-facility transports	1733 (11.0%)	160 (12.0%)	29 (7.5%)	
Self-referral	1598 (10.1%)	125 (9.4%)	33 (8.5%)	
Intra-hospital infarct	399 (2.5%)	36 (2.7%)	5 (1.3%)	
Clinical data				
Hypertension	9245 (59%)	791 (60%)	229 (59%)	0.903
Diabetes mellitus	2842 (18%)	264 (20%)	79 (20%)	0.812
Previous angina pectoris	1604 (10%)	147 (11%)	31 (8%)	0.083
Hyperlipidemia	4385 (28%)	372 (28%)	119 (31%)	0.291
Family history of cardiovascular events	2979 (19%)	225 (17%)	74 (19%)	0.317
Current smoker	6380 (40%)	523 (39%)	155 (40%)	0.805
Previous myocardial infarction	1793 (11%)	153 (12%)	47 (12%)	0.733
Previous stroke	681 (4%)	53 (4%)	17 (4%)	0.723
Previous angioplasty	1936 (12%)	160 (12%)	58 (15%)	0.125
Previous CABG	354 (2%)	28 (2%)	10 (3%)	0.575
Renal failure	779 (5%)	61 (5%)	26 (7%)	0.093
Out-of-hospital cardiac arrest	1461 (9%)	127 (10%)	41 (11%)	0.545
Cardiogenic shock	1984 (13%)	169 (13%)	50 (13%)	0.916
TIMI risk score	3.89 ± 0.02	3.88 ± 0.07	3.87 ± 1.31	0.464
ECG (STEMI site) (*n* = 15,694)				
Anterior	7044 (45%)	601 (46%)	176 (46%)	0.978
Inferior	7414 (47%)	603 (46%)	175 (46%)	
Lateral	965 (6%)	83 (6%)	22 (6%)	
LBBB	271 (2%)	32 (2%)	10 (3%)	
Thrombolytic therapy (*n* = 15,800)	122 (0.8%)	10 (0.8%)	3 (0.8%)	0.964
Primary PCI treatment (*n* = 15,800)	14,186 (89.8%)	1,205 (90.7%)	352 (91.0%)	0.864
Hospital mortality (*n* = 15,768)	1388 (8.8%)	118 (8.9%)	37 (9.6%)	0.680
Angiographic results				
No. coronary arteries narrowed (*n* = 15,388)				
0	588 (4%)	47 (4%)	11 (3%)	0.382
1	5185 (34%)	447 (34%)	129 (35%)	
2	4538 (29%)	367 (28%)	120 (32%)	
3	4981 (32%)	431 (33%)	110 (29%)	
LMCA	96 (0.6%)	5 (0.4%)	3 (0.8%)	
CTO in NIRA (*n* = 15,733)	1802 (12%)	156 (12%)	44 (11%)	0.829
Recanalized vessel (*n* = 14,163)				
LAD	6271 (40%)	541 (45%)	154 (44%)	0.521
RCA	5685 (36%)	480 (40%)	136 (39%)	
LCX	1939 (12%)	165 (14%)	52 (15%)	
LMCA	179 (1%)	10 (1%)	6 (2%)	
Graft	89 (1%)	8 (1%)	4 (1%)	
TIMI angiographic flow grade before PCI (*n* = 14,469)				
Score 0–2	13,284 (92%)	1138 (92)	325 (91%)	0.358
Score 3	1185 (8%)	92 (7%)	32 (9%)	
TIMI angiographic flow grade after PCI (*n* = 14,174)				
Score 0–2	876 (6%)	78 (6%)	20 (6%)	0.591
Score 3	13,298 (94%)	1127 (94%)	332 (94%)	

Data are presented as percentages or means and standard errors of the mean for the total study cohort. Angiographic data refer to patients in whom an angiogram was performed. A comparison between the subgroups of patients treated in March 2020 at the peak of the COVID-19 crisis during the German lockdown and the preceding three March months from 2017 to 2019 is included

*CABG* coronary artery bypass grafting, *CTO* chronic total occlusion, *ECG* electrocardiogram, *EMS* transport by emergency medical service, *LAD* left anterior descending artery, *LBBB* left bundle branch block, *LCX* left circumflex artery, *LMCA* left main coronary artery, *NIRA* non-infarct-related arteries, *PCI* percutaneous coronary intervention, *RCA* right coronary artery, *SD* standard deviation, *TIMI* Thrombolysis in Myocardial Infarction

### Altered admission modes at the peak of the COVID-19 crisis

**Fig. 2:**
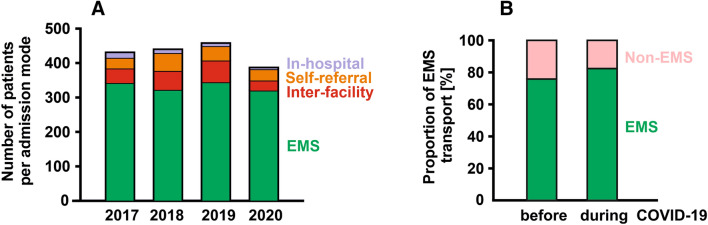
Modes of admission to hospitals for STEMI patients during the corona virus crisis. **a** Total numbers of STEMI patients admitted to PCI hospitals by the indicated modes in March 2020 compared to the March months in the three preceding years 2017–2020. **b** Increased percentage of patients admitted by emergency medical service (EMS) during the COVID-19 outbreak in March 2020 as compared to the previous March months in 2017–2019, whereas the proportion of non-EMS transports was reduced

When the COVID-19 crisis peaked in Germany in March 2020, there were 387 documented STEMI cases treated in the centres of the FITT-STEMI cluster, which corresponds to a decline of 12.6% as compared to the March months of the previous 3 years (Table 1). In addition, we noted significant changes in the distinct transport modes to hospital admission (*p* = 0.017) (Fig. 2a). The most prominent decline in the proportion of case numbers was found for STEMI patients suffering from in-hospital infarction (2.7% vs. 1.3%) followed by patients transported indirectly from external, non-PCI-capable centres (12.0% vs. 7.5%), because the majority of hospitals had previously discharged all their elective patients due to the general shutdown associated with the COVID-19 crisis. Likewise, the percentage of self-referred STEMI patients was reduced in the last March month (9.4% vs. 8.5%), whereas the percentage of patients transported directly from the pre-hospital setting to the hospital by EMS increased from 75.8% to 82.7% (Fig. 2b). Since this group of STEMI patients who called the EMS to seek medical help because of ischaemia-induced chest pain always constituted the majority of STEMI cases, the total number of 387 STEMI patients treated in the March 2020 during the crisis was only slightly smaller than the monthly case number averaged from the March months 2017–2019 (Fig. 2a).

### Stability of key quality indicators for STEMI care during the SARS-CoV-2 outbreak

**Fig. 3:**
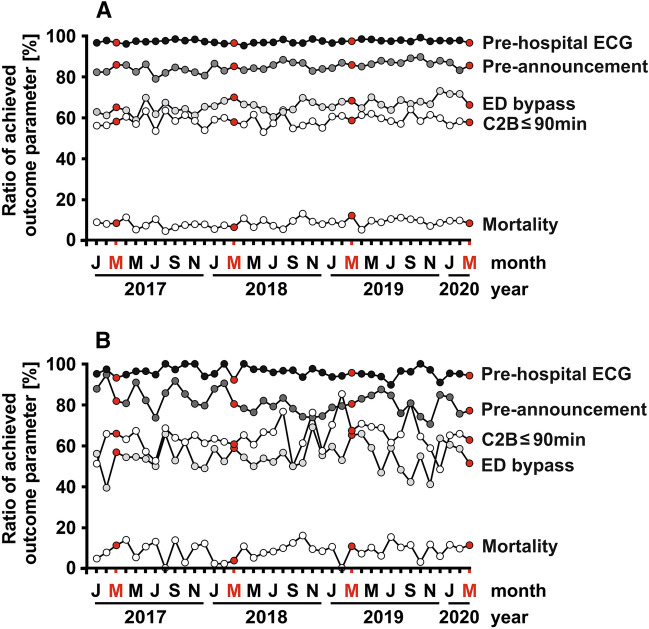
Percentages of achieved key quality indicators for STEMI treatment over the 39-month period from January 2017 to March 2020 in all the 41 participating FITT-STEMI study centres (**a**) and a subgroup thereof with seven centers in the Lower Rhine area heavily engaged in fighting the SARS-CoV-2 pandemic (**b**). March months are indicated by the red letter “M”. The last point in each curve corresponds to the lockdown month with high COVID-19 case numbers (March 2020)

The total number of EMS-transported patients treated within 360 min after first medical contact was reduced when compared to the mean of monthly cases in the preceding three March months (*n* = 270 patients vs. *n* = 904 patients, − 10.4%) (Table 2). When assessing key quality indicators, we found that pre-hospital ECGs were recorded for 261 of these 270 EMS-transported patients (96.7%, Table 2, Fig. 3a). This percentage was stable, when compared to the three previous COVID-19-free March months (96.9%, 95%CI = − 2.2–2.7, *p* = 0.846). Similarly, the number of direct transfers to PCI treatment bypassing the emergency department remained at the same high level, as *n* = 179 (66.3%) patients reached the catheterization laboratory without a time-consuming delay before the reperfusion therapy. In the three preceding March months, the percentage of patients arriving at the catheterization laboratory without a stop at the emergency department was not significantly changed (67.8%, 95% CI = − 4.9–7.9, *p* = 0.641). In addition, there was no significant difference in the proportion of STEMI patients with treatment times from first medial contact to balloon inflation of equal or less than 90 min (57.8% vs. 58.3%, 95%CI = − 6.2–7.2, *p* = 0.879). Except for longer transport times from the field to the hospital (*p* = 0.003), the seven university clinics did not differ from other tertiary hospitals with respect to treatment times and quality indicators for STEMI care during the lockdown.

**Table 2:** Changes in reported key quality indicators for EMS-transported and PCI-treated STEMI patients who had treatment times from first medical contact to balloon inflation of equal to or less than 360 min

	Total study cohort (*n* = 10,633)	Patients treated in the March months 2017–2019 (*n* = 904)	Patients treated in the March month 2020 (*n* = 270)	Difference [95%-confidence interval]	*P* value
Symptom-to-contact time (min) (*n* = 10,455)	159.1 ± 2.3	163.1 ± 7.9	150.4 ± 13.6	12.7 [− 19.1; 44.5]	0.433
Duration of EMS at scene (*n* = 10,514)	23.1 ± 0.1	23.1 ± 0.4	22.5 ± 0.8	0.6 [− 1.1; 2.3]	0.502
Transport time by EMS (*n* = 10,512)	16.8 ± 0.1	16.7 ± 0.3	17.0 ± 0.6	− 0.2 [− 1.5; 1.0]	0.705
Contact-to-door time (min) (*n* = 10,629)	39.9 ± 0.2	39.9 ± 0.5	39.4 ± 1.0	0.5 [− 1.6; 2.7]	0.629
Door-to-cath time (min) (*n* = 10,608)	19.8 ± 0.3	18.7 ± 0.9	18.9 ± 1.7	− 0.2 [− 4.0; 3.7]	0.925
Cath-to-puncture time (min) (*n* = 10,572)	13.2 ± 0.1	12.9 ± 0.3	14.1 ± 0.5	− 1.2 [− 2.3; − 0.1]	0.029
Puncture-to-balloon (min) (*n* = 10,612)	20.4 ± 0.1	19.7 ± 0.4	20.2 ± 0.8	− 0.47 [− 2.21; 1.27]	0.594
Door-to-balloon time (min) (*n* = 10,629)	53.0 ± 0.3	51.3 ± 1.1	53.2 ± 2.0	− 1.9 [− 6.3; 2.6]	0.407
Contact-to-balloon time (min) (*n* = 10,633)	92.9 ± 0.4	91.3 ± 1.2	92.6 ± 2.2	− 1.3 [− 6.2; 3.5]	0.592
ECG before arrival at the hospital (*n* = 10,633)	10,348 (97%)	876 (97%)	261 (97%)	0.2 [− 2.2; 2.7]	0.846
Pre-announcement by telephone (*n* = 10,633)	9,037 (85%)	774 (86%)	231 (86%)	0.1 [− 4.7; 4.8]	0.979
Emergency department bypass (*n* = 10,633)	7,016 (66%)	613 (68%)	179 (66%)	1.5 [− 4.9; 7.9]	0.641
Contact-to-balloon time ≤ 90 min (*n* = 10,633)	6,235 (58.6%)	527 (58.3%)	156 (57.8%)	0.5 [− 6.2; 7.2]	0.879
Hospital mortality (*n* = 10,633)	914 (8.6%)	83 (9.2%)	23 (8.5%)	0.7 [− 3.2; 4.5]	0.739

Data are given as numbers and percentages or means and standard errors of the mean

### Pre- and intra-hospital treatment times during the COVID-19 crisis

We found no evidence that symptomatic STEMI patient waited longer to seek medical help during the virus outbreak for fear of an hospital-acquired infection, as the mean time interval from symptom onset to first medical contact was not prolonged (163.1 ± 7.9 min vs. 150.4 ± 13.6 min, 95% CI = − 19.1–44.5, *p* = 0.433). For all STEMI patients transported by EMS and treated within 360 min after first medical contact, the mean contact-to-door time in the March months from 2017–2019 was 39.9 ± 0.5 min (Table 2, Fig. 3). In March 2020, this time interval was in a similar range (39.4 ± 1.0 min), indicating that there were no statistically significant and clinically relevant delays during the COVID-19 precautions (95% CI = − 1.6–2.7, *p* = 0.629). Likewise, the mean door-to-balloon time was similar in the last March month to the three preceding ones (51.3 ± 1.1 min vs. 53.2 ± 2.0 min, 95% CI = − 6.3–2.6, *p* = 0.407) (Fig. 3). In all, the treatment times from arrival at the field to balloon inflation during PCI did not significantly differ between the two groups (91.3 ± 1.2 min vs. 92.6 ± 2.2 min, 95% CI = − 6.2–3.5, *p* = 0.592). Regression models adjusted for gender, age, TIMI risk score, infarct localization, and thrombolytic therapy confirmed these results (Supplemental Table 1). However, in univariate analysis, there was a small but significant difference in the mean times from arrival at the catheterization laboratory to vessel puncture between patients treated before or during the COVID-19 outbreak (12.9 ± 0.3 min vs. 14.1 ± 0.5 min, 95% CI = − 2.3–-0.1, *p* = 0.029).

### Revascularization rates and in-hospital mortality during the COVID-19 crisis

Angiographic results showed a similarly high rate of complete revascularization in patients treated during the outbreak of the SARS-CoV-2 infections as compared to the success rate of reperfusions in the three March months before. As measured by a TIMI grade 3 coronary flow, a complete antegrade perfusion into the vessel bed distal to the obstruction was achieved in more than nine out of ten patients in the two groups (93.5% vs. 94.3%, *p* = 0.591) (Table 1). At the peak of the COVID-19 outbreak, the mortality rate did not differ from the period before the viral outbreak, neither in the sample of all STEMI patients (Table 1) nor in the subgroup of EMS-transported, PCI-treated patients (Table 2). In March 2020, there were 23 deaths (8.5%) in EMS-transported STEMI patients in whom the PCI procedure was performed within 360 min of first medical contact, while there were on average 27.7 monthly deaths (9.2%) in the March months of the three preceding years (95% CI = − 3.2–4.5, *p* = 0.739) (Table 2, Fig. 3).

### Subgroup analysis of clinics heavily affected by the SARS-CoV-2 outbreak

**Fig. 4:**
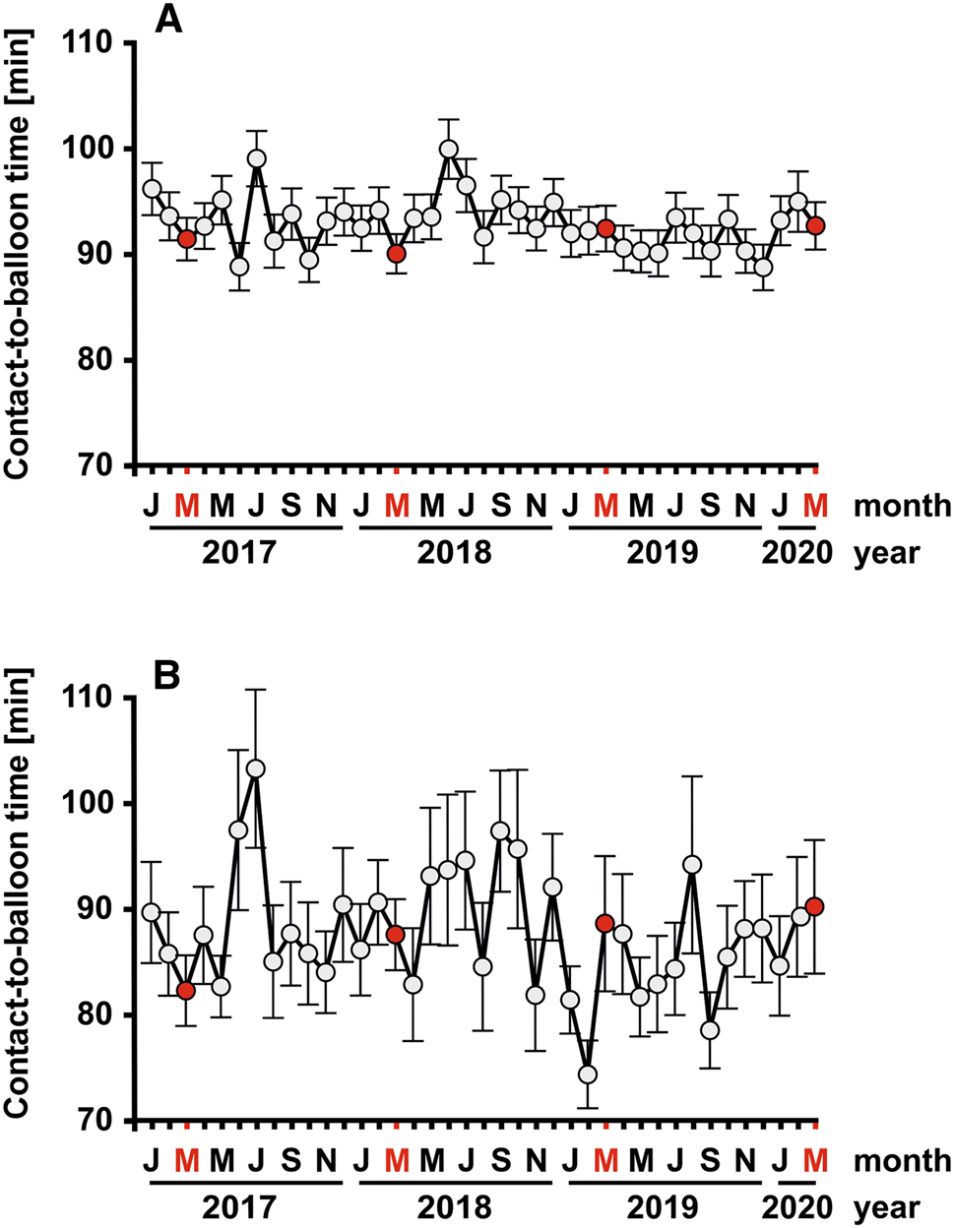
Monthly contact-to-balloon times, as presented by their means and standard errors of the mean, in STEMI patients treated within 360 min from first medical contact to balloon inflation in the total study group (**a**) and the subgroup of clinics in the Lower Rhine area with high case numbers of COVID-19 (**b**). The last point in each curve corresponds to the lockdown March month 2020 with a high prevalence of COVID-19 in Germany

The Lower Rhine area around Heinsberg in North Rhine-Westphalia had emerged as a hot spot for the spread of the coronavirus with the highest numbers of SARS-CoV-2-positive cases in Germany, particularly in the initial phase of the virus outbreak (Fig. 1). In a subgroup analysis of seven FITT-STEMI clinics located in the centre of this heavily affected area, we found that four key quality indicators for STEMI care stayed stable and did not deteriorate during the course of the infection (Table 3, Fig. 3b). The rate of pre-hospital ECG recordings was unaltered, as only two out of 35 cases (94.3%) arrived at the PCI hospital without an ECG-based diagnosis of myocardial infarction compared to nine out of 141 patients in the three previous March months (93.6%). This difference in the number of ECG recordings was not statistically significant (95% CI = − 9.4–8.0, *p* = 0.884). Similarly, neither was the number of direct transports to the catheterization laboratory bypassing the emergency department (60.3% vs. 51.4%, 95% CI = − 9.6–27.3, *p* = 0.341) nor the proportion of contact-to-balloon times within 90 min (64.5% vs. 62.9%, 95% CI = − 19.5–16.2, *p* = 0.853) reduced significantly at the peak of the coronavirus outbreak. Likewise, the proportion of pre-announcement by telephone before the ambulance arrived at the hospital did not differ between the groups (80.9% vs. 77.1%, 95% CI = − 11.6–19.1, *p* = 0.623). Except for a shorter EMS transport time by 3 min during the lockdown (15.1 ± 0.7 min vs 12.1 ± 1.2 min, 95% CI = 0.04–5.90, *p* = 0.047), all other measured time intervals did not differ when compared for the months with and without infections (Table 3, Fig. 4b).

**Table 3:** Subgroup analysis of key quality indicators for STEMI management in seven FITT-STEMI clinics in the Lower Rhine areal located in a hot spot of COVID-19 outbreak during the pandemic as compared to the three preceding March months

	STEMI patients treated in the Lower Rhine area (*n* = 1538)	Patients treated in the March month 2017/2018/ 2019 (*n* = 141)	Patients treated in March month 2020 (*n* = 35)	95%-confidence interval	*P* value
Symptom-to-contact time (min) (*n* = 1512)	155.1 ± 6.1	178.1 ± 22.2	105.4 ± 23.1	72.7 [− 17.5; 162.8]	0.113
Duration of EMS at scene (*n* = 1502)	21.8 ± 0.3	22.1 ± 0.9	21.7 ± 2.4	0.3 [− 3.8; 4.5]	0.873
Transport time by EMS (*n* = 1502)	14.7 ± 0.2	15.1 ± 0.7	12.1 ± 1.2	2.97 [0.04; 5.90]	0.047
Contact-to-door time (min) (*n* = 1538)	36.6 ± 0.3	37.5 ± 1.1	33.9 ± 2.5	3.6 [− 1.4; 8.7]	0.190
Door-to-cath time (min) (*n* = 1536)	19.8 ± 1.1	18.2 ± 2.5	23.4 ± 6.0	− 5.2 [− 16.7; 6.3]	0.376
Cath-to-puncture time (min) (*n* = 1533)	13.3 ± 0.2	13.4 ± 0.6	14.0 ± 1.3	− 0.6 [− 3.5; 2.2]	0.657
Puncture-to-balloon (min) (*n* = 1538)	19.0 ± 0.3	17.2 ± 0.7	18.9 ± 1.5	− 1,7 [− 4.8; 1.35]	0.258
Door-to-balloon time (min) (*n* = 1538)	51.1 ± 0.8	48.7 ± 2.5	56.3 ± 6.0	− 7.6 [− 19.2; 4.0]	0.200
Contact-to-balloon time (min) (*n* = 1538)	87.6 ± 0.8	86.3 ± 2.6	90.2 ± 6.3	− 3.9 [− 16.0; 8.2]	0.524
ECG before arrival at the hospital (*n* = 1538)	1474 (95.8%)	132 (93.6%)	33 (94.3%)	− 0.6 [− 9.4; 8.0]	0.884
Pre-announcement by telephone (*n* = 1538)	1253 (81.5%)	114 (80.9%)	27 (77.1%)	3.7 [− 11.6; 19.1]	0.623
Emergency department bypass (*n* = 1538)	847 (55.1%)	85 (60.3%)	18 (51.4%)	8.9 [− 9.6; 27.3]	0.341
Contact-to-balloon time ≤ 90 min (*n* = 1538)	991 (64.4%)	91 (64.5%)	22 (62.9%)	1.7 [− 19.5; 16.2]	0.853
Hospital mortality (*n* = 1538)	135 (8.8%)	12 (8.5%)	4 (11.4%)	− 2.9 [− 14.4; 8.6]	0.591

Data and patients included are given as numbers or means and percentages or standard errors of the mean including the 95%-confidence interval for EMS-transported and PCI-treated STEMI patients with contact-to-balloon times reported equal to or less than 360 min

## Discussion

By analysing the impact of the SARS-CoV-2 pandemic on STEMI treatment in established networks, we noted four important points. Firstly, we found only a mild decrease in the absolute number of STEM patients treated in systems of STEMI care in Germany (12.6%). Secondly, this decrease included STEMI patients with self-referral, transfer patients from non-PCI hospitals, and patients suffering myocardial infarction during hospital stay, whereas the number of EMS-transported STEMI cases remained stable. Thirdly, except for a significantly prolonged time from arrival at the catheterization laboratory to vessel puncture, there were no significant changes in parameters of STEMI care and time lines of the treatment chain in the group of STEMI patients directly brought by EMS to the PCI-hospitals. Fourthly, even in the hot spot of SARS-CoV-2 infections during March 2020 in the heavily affected Lower Rhine area, which was completely covered by hospitals involved in the FITT-STEMI programme, we found no impairment in the established quality indicators for STEMI care.

In Germany, STEMI patients transported by EMS from the scene constitute the vast majority of acute PCI-treated STEMI cases and this number did not decline during the virus outbreak. Although the case numbers of patients with self-referral or inter-facility transports were remarkably reduced, there was only a moderate decrease in total STEMI numbers at the peak of the infections. This finding is in contrast to three recent reports on decreasing numbers of STEMI treatment published from cardiac catheterization laboratories in the United States and Spanish PCI centres [8, 20, 21].

Notably, in our cohort, there were neither significant delays in the contact-to-door nor the door-to-balloon time related to the outbreak of the virus. However, owing to safety precautions in preparation for the PCI procedure, the time interval from arrival at the catheterization laboratory to vessel puncture was significantly prolonged. This observation indicates that the interventional cardiologists took concerns seriously having been raised about the risk of peri-procedural transmission of SARS-CoV-2 between STEMI patients and medical staff as well as a further dissemination of the disease [8, 21, 22]. It has been assumed that the precautions and prioritization when taking care of patients with confirmed or possible COVID-19 may prolong treatment times, thereby affecting the quality of STEMI treatment [7]. However, we demonstrate here that these precaution procedures did not result in a significant delay in reperfusion therapy.

Other process-relevant and clinically important indicators of STEMI care performance were also not impaired, such as the proportion of pre-hospital ECG recordings, direct transports to the catheterization laboratory bypassing the emergency department and the achievement of the guideline-recommended contact-to-balloon time of less than 90 min. These data from Germany are not in line with a recent report from a small sample of seven STEMI patients treated in Hong Kong during the COVID-19 pandemic, which showed dramatic changes in treatment times [7]. A report from Italy demonstrated that the cumulative incidence of out-of-hospital cardiac arrest was associated with the cumulative incidence of SARS-CoV-2 infections and that the EMS arrival time was increased during the virus outbreak [23].

In the FITT-STEMI programme, feedback mechanisms had been successfully implemented to shorten treatment times and improve prognosis in STEMI patients in previous years [2, 17, 18, 24]. Even when the EMS systems and the hospitals were faced with the burden of the coronavirus pandemic, the pre-established routine pathways in STEMI care were maintained throughout the crisis and resulted in a stable high proportion of patients achieving the guideline-recommended 90-min limit from first medical contact to reperfusion. Thus, the successful implementation of the requirements for high-standard STEMI treatment already before the virus outbreak may have saved the participating FITT-STEMI centres from significant treatment delays, despite the rapidly increasing numbers of infections.

Even clinics confronted with high case numbers of SARS-CoV-2-infected patients, such as the hospitals in the Lower Rhine area, were capable of maintaining high process standards in their routine STEMI management. Our measurements were sensitive enough to detect small changes in treatment times, as there was a significant 3-min shortening during the virus outbreak from the scene to the hospital, which probably reflects the slowdown of traffic at that time, thereby promoting road-based transportation. Despite their fight against the spread of the virus, the hot-spot hospitals in the Heinsberg area preserved their obligations for protocol-based STEMI care using predefined quality markers for timely reperfusion. Therefore, concerns about a limited number of interventional cardiologists and expert medical personnel available in the catheterization laboratory during the outbreak period proved to be unfounded.

When faced by the challenge of SARS-CoV-2-positive patients or subjects with an unknown viral status, some authors reconsidered intravenous thrombolysis as an alternative, albeit less effective strategy for coronary revascularization [21, 25, 26]. However, our data have indicated that in pre-existing functional STEMI care networks the success rate for PCI treatment remains at a high level, suggesting that there is no need to recommend fibrinolytic therapy and abandon PCI.

Several issues merit consideration in the interpretation of the present results from the observational FITT-STEMI study. Firstly, the registry-based, cross-sectional nature of the FITT-STEMI trial may be susceptible to unmeasured confounding and selection bias, such as non-system reasons for treatment delays independent of the coronavirus crisis. Secondly, other German PCI hospitals, which are not members in the FITT-STEMI consortium, may be less privileged in their treatment pathways for STEMI patients, because they do not receive regular site-specific data assessments on treatment times and in-hospital mortality using interactive feedback sessions. Therefore, it is formally not possible to extrapolate our findings to all German PCI centres. Thirdly, we assessed only short-term outcome, as data on long-term follow-up are currently not available and the SARS-CoV-2 outbreak is still not under control. Fourthly, the findings from our study conducted in Germany are not applicable to STEMI management care systems in other countries, in which exclusively paramedics are part of the EMS transportation teams and not physicians experienced in emergency medicine. Thus, our results cannot be generalized to STEMI care systems in other countries using different pathways for treating their patients.

In summary, during the COVID-19 crisis, we observed no impairment in the quality of STEMI care in the German FITT-STEMI hospitals. Our data suggest that a pre-established, robust organisational structure for STEMI care is an important requirement for timely treatment of STEMI patients and ensures the stability of outcome-relevant performance parameters, even when confronted with the burden of the global, ongoing pandemic.

## Electronic supplementary material

Below is the link to the electronic supplementary material.

Supplementary file1 (DOCX 34 kb)
