# Supplementary material for: Impact of COVID-19 outbreak on regional STEMI care in Germany
Source: Clin Res Cardiol. 2020 Jul 16;109(12):1511–21. doi: 10.1007/s00392-020-01703-z (PMC7364412; doi:10.1007/s00392-020-01703-z)
Supplement: Supplementary file 1 — Supplementary file1 (DOCX 34 kb) [file 392_2020_1703_MOESM1_ESM.docx]

Supplemental Table 1: Results from a series of linear regression models with the indicated time intervals as dependent variables and study recruitment in the March months in the three preceding years versus March 2020 during the corona virus outbreak as independent variable. All models were adjusted for gender, age, TIMI risk score, infarct localization, and thrombolytic therapy. Logistic regression models for key quality markers in STEMI care as independent, dichotomous variables were created using the same set of covariables (n=1,174).

|  | n | Regression coefficient B | 95%-confidence interval | P value |
| --- | --- | --- | --- | --- |
| Symptom-to-contact time | 1,127 | 17.833 | [-14.037; 49.703] | 0.272 |
| Time of EMS at scene | 1,133 | 0.770 | [-0.873; 2.413] | 0.358 |
| Transport time by EMS | 1,133 | -0.236 | [-1.528; 1.057] | 0.721 |
| Contact-to-door time | 1,147 | 0.700 | [-1.408; 2.808] | 0.515 |
| Door-to-cath time | 1,145 | 0.310 | [-3.579; 4.200] | 0.876 |
| Cath-to-puncture time | 1,142 | -1.127 | [-2.213; -0.041] | 0.042 |
| Puncture-to-balloon time | 1,146 | -0.358 | [-2.104; 1.388] | 0.687 |
| Door-to-balloon time | 1,147 | -1.173 | [-5.563; 3.217] | 0.600 |
| Contact-to-balloon time | 1,147 | -0.472 | [-5.224; 4.279] | 0.845 |

|  | n | Odds ratio | 95%-confidence interval | P value |
| --- | --- | --- | --- | --- |
| Pre-hospital ECG | 1,147 | 0.964 | [0.438; 2.119] | 0.927 |
| Pre-announcement | 1,147 | 0.965 | [0.652; 1.429] | 0.860 |
| ED bypass | 1,147 | 1.035 | [0.772; 1.386] | 0.820 |
| Contact-to-balloon time ≤90 min | 1,147 | 0.982 | [0.741; 1.301] | 0.898 |
| Hospital mortality | 1,147 | 1.200 | [0.692; 2.083] | 0.516 |
